# Atypical myeloproliferative neoplasm with concurrent *BCR-ABL1* fusion and *CALR* mutation: A case report and literature review

**DOI:** 10.1097/MD.0000000000018811

**Published:** 2020-01-31

**Authors:** Chunshui Liu, Ruiping Hu, Zhonghua Du, Manuel Abecasis, Cong Wang

**Affiliations:** a Department of Hematology, The First Hospital of Jilin University, Changchun, Jilin, China; b Department of Hematology, Instituto Português de Oncologia de Francisco Gentil, Lisbon, Portugal.

**Keywords:** *BCR-ABL1* fusion, *CALR* mutation, chronic myelogenous leukaemia, clonal evolution, cytogenetic response

## Abstract

**Rationale:**

Concurrent calreticulin (*CALR*) mutation and *BCR-ABL1* fusion are extremely rare in chronic myelogenous leukemia; to date, only 12 cases have been reported.

**Patient concerns:**

A 57-year-old male who had an 11-year history of essential thrombocytosis presented to our hospital with leukocytosis and marked splenomegaly for 3 months.

**Diagnoses:**

Chronic myelogenous leukemia with myeloid fibrosis arising on the background of essential thrombocytosis harboring both *BCR-ABL1* fusion and type-1 like *CALR* mutation.

**Interventions:**

Imatinib was started at 300 mg daily and increased to 400 mg daily after 3 months; interferon was added after 12 months.

**Outcomes:**

Partial cytogenetic response was achieved after 3 months of imatinib therapy and complete cytogenetic response was achieved after 1 year of treatment. However, *CALR* mutation was still present with a stable mutational allele burden.

**Lessons:**

In this case report and review of additional 12 cases with simultaneous presence of *CALR*-mutation and *BCR-ABL1* fusion, we highlighted the importance of integrating clinical, morphological, and molecular genetic data for classifying atypical myeloid neoplasms.

## 1 Introduction

Myeloproliferative neoplasms (MPNs) are caused by mutations of various genes, resulting in constitutive activation of their corresponding signaling transduction pathways and aberrant hematopoiesis. Mutation in a gene or a group of genes consequently leads to particular phenotypes and clinical manifestations of MPNs.^[1]^ Key genetic aberrations identified to date include *BCR-ABL1* rearrangement in Philadelphia chromosome-positive (Ph^+^) chronic myelogenous leukemia (CML) and mutations of Janus kinase 2 (*JAK2*)/myeloproliferative leukemia protein (*MPL*)/calreticulin (*CALR*) in Philadelphia chromosome-negative (Ph^–^) MPNs.^[1,2]^ These gene alterations were initially thought to be mutually exclusive,^[3,4]^ although recent case reports have shown the simultaneous presence of these mutations in MPNs.^[5–7]^

Mutations of *CALR* have been reported primarily in wild-type *JAK2* and *MPL*-related essential thrombocytosis (ET) and primary myelofibrosis (PMF). More recently, mutations in *CALR* exon 9 have been reported to occur in 20% to 25% of ET cases and 25% to 35% of PMF cases,^[2,4]^ but *CALR* mutations are extremely rare in MPNs with the t(9;22)/*BCR-ABL1*; to date, only 12 cases have been reported.

In the present case report, we describe a CML patient with an 11-year history of ET who harbored both *BCR-ABL1* fusion and type 1-like *CALR* mutation, a previously unreported atypical MPN case. We then systematically reviewed 12 MPN cases reported in the literature and summarized their characteristics, clonal evolution, treatment options, and responses.

## 2 Case presentation

**Figure 1:**
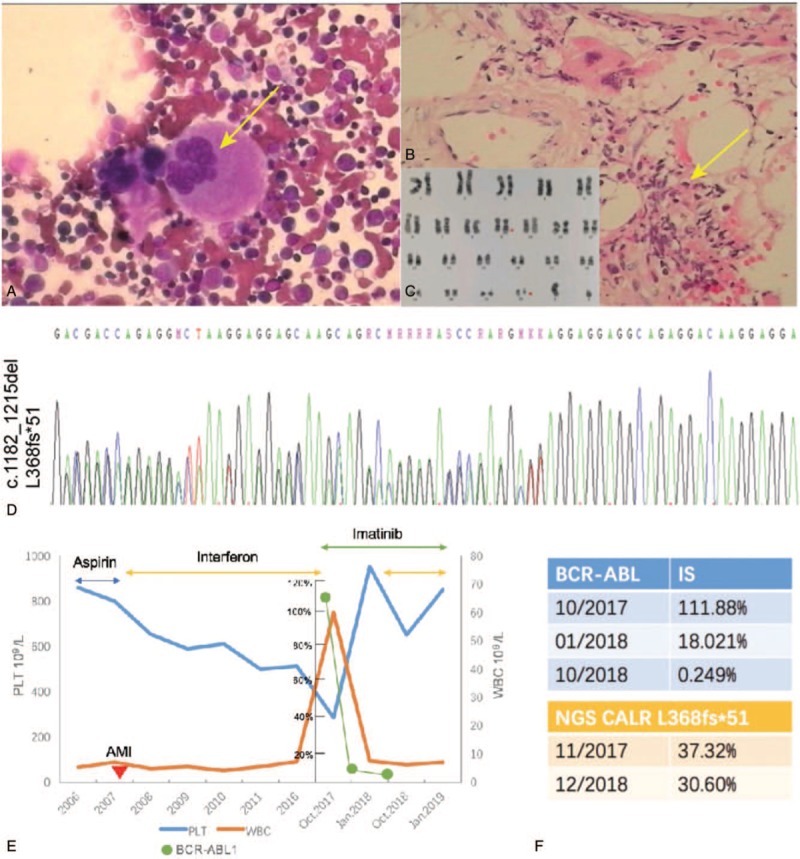
. Clinicopathological features of atypical myeloproliferative neoplasm with *BCR-ABL1* fusion and calreticulin (*CALR*) mutation. (A) Bone marrow aspiration and Wright–Giemsa staining. The patient was admitted into our hospital and underwent bone marrow aspiration with Wright–Giemsa staining. The data show enlarged megakaryocytes and hyperlobulated nuclei, but not classical “dwarf” morphology in megakaryocytes (original magnification, ×400). (B) Hematoxylin and eosin staining of the bone marrow biopsy specimen. The patient was followed up in 2017, and bone marrow biopsy was repeated. Hematoxylin and eosin staining shows leukocytosis and severe marrow fibrosis (original magnification, ×400). (C) Karyotyping of the bone marrow. The data show 46,XX,t[9;22](q34;q11)[10]. (D) Sanger sequencing. The bone marrow was subjected to polymerase chain reaction (PCR) using the primers 5′-GTGGGGCGTAACAAAGGTGA-3′ and 5′-AGAGACATTATTTGGCGCGG-3′ and Sanger sequencing. The data show a frameshift mutation of *CALR* exon 9 hotspot (c.1182-1215delaaggaggaggaagaagacaagaaacgcaaagagg, p.L368fs^∗^51). (E) Graphic depiction of peripheral blood counts from 2006 to January 2019. The patient was treated initially with interferon after acute myocardial infarction from 2007 to 2017, and the platelet count was sustained around 400 to 600 × 109/L. In October 2017, he was switched to imatinib due to chronic myeloid leukemia based on leukocytosis, splenomegaly, and *BCR-ABL1* fusion. After 3 months of imatinib treatment, thrombocytosis had worsened even though the *BCR-ABL1* transcript levels markedly decreased. To control the platelet count, the patient was given a combination of imatinib with interferon. (F) Quantitative reverse-transcriptase (qRT)-PCR. The bone marrow samples were subjected to qRT-PCR (the top table) and next-generation DNA sequencing (the bottom table). The qRT-PCR data show the level of *BCR-ABL* fusion transcript as an international standard value, whereas the bottom table shows the allele burden of *CALR* mutation.

In 2006, a 46-year-old male visited our out-patient clinic in The First Hospital of Jilin University (Changchun, China). His laboratory tests showed a platelet count of 800 × 10^9^/L with a normal white blood cell count and hemoglobin level. A bone marrow aspiration smear showed enlarged megakaryocytes with hyperlobulated nuclei and without the classical “dwarf” morphology (Fig. 1A). Bone marrow biopsy further revealed an increase in megakaryocytes, but with normocellular morphology and a normal myeloid-to-erythroid ratio, suggesting a diagnosis of ET without reticulin and collagen fibrosis. The genetic test showed wild-type JAK2 V617F expression at that time. Aspirin was prescribed, and 1 year later, the patient experienced acute myocardial infarction for which 2 stents were placed in his left anterior descending coronary artery. After recovery from the percutaneous coronary intervention, he was given interferon, and his platelet count reached 400 to 600 × 10^9^/L.

The disease remained stable until 2017 when he displayed marked splenomegaly (19.1 cm in length) and leukocytosis. Thus, he was referred to our in-patient services. Laboratory tests revealed a leukocyte count of 69.88 × 10^9^/L and platelet count of 285 × 10^9^/L. Bone marrow aspiration and biopsy showed a significantly increased myeloid-to-erythroid ratio with 3% myeloblasts and severe marrow fibrosis, respectively (Fig. 1B). Karyotyping showed 46,XX,t[9;22](q34;q11)[20] (Fig. 1C). Quantitative reverse-transcriptase polymerase chain reaction data demonstrated positive *BCR-ABL p210* fusion gene, while the quantitative result was 111.88% of an international scale (IS) value. DNA sequencing data also confirmed the presence of type 1-like *CALR* mutation (c.1182_1215del, L368fs∗51) (NM_004343.3) (Fig. 1D) with a mutational allele burden of 37.32% (Fig. 1F). Thereafter, he was diagnosed with CML in a chronic phase with myeloid fibrosis, probably arising on the background of ET. The treatment regimen was switched from interferon to tyrosine kinase inhibitor (TKI) imatinib at 300 mg daily. Three months later, he achieved a partial cytogenetic response: the Ph+ positive rate decreased to 5% and the *BCR-ABL1* fusion gene rate declined to 18.02% (IS) (Fig. 1F). Bone marrow biopsy showed a normal myeloid-to-erythroid ratio, but an increase in large megakaryocytes with grade 3 fibrosis. Imatinib dose was increased to 400 mg daily. One year later, the size of his spleen had obviously shrunk and he achieved a complete cytogenetic response with Ph-negativity and a decrease in the *BCR-ABL1* fusion gene rate to 0.249% (IS). However, his platelet count was 900 × 10^9^/L and *CALR* mutation was still present with a mutational allele burden of 30.60% (Fig. 1F). Bone marrow biopsy still showed grade 3 fibrosis. To further control the platelet count, he was given a combination of imatinib and interferon, and his follow-up showed stable disease. This patient's disease process is summarized and shown in Figure 1E.

## 3 Discussion

In this article, we reported our own case and then performed literature search to identify additional MPN cases that harbored both *CALR* mutation and *BCR-ABL1* fusion. We identified 12 cases from PubMed. The details, such as clinical characteristics, clonal evolution, treatment options, and responses of our patient and these 12 cases, are summarized in Table 1 and discussed below.

**Table 1:**
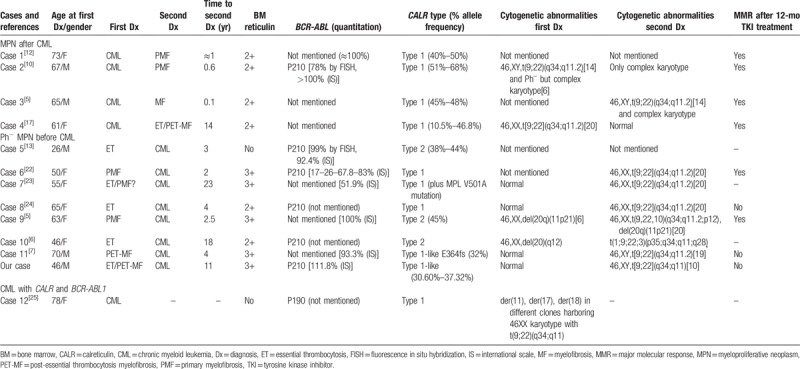
Clinical and molecular characteristics of 13 MPN patients with *BCR-ABL1* fusion and CALR mutation.

### 3.1 Clinical characteristics

Overall, this cohort of 13 patients had a median age of 63 years (range: 26–78 years; Table 1) at the initial diagnosis, and 5 were male for a male/female ratio of 1:1.6 (Table 1). Interestingly, most of the patients were diagnosed with PMF or post-essential thrombocythemia myelofibrosis, either before or after CML diagnosis, suggesting that reticulin fibrosis was the most common phenomenon in patients with concurrent *CALR* mutation and *BCR-ABL1* fusion.

In this cohort, patients were all diagnosed with chronic phase CML, and 8 of 13 patients were diagnosed with Ph^−^ MPN (such as PMF, ET, or post-essential thrombocythemia myelofibrosis) before CML diagnosis. There was a shift to the clinical BCR-ABL1^+^ CML phenotype, with manifestations such as splenomegaly and leukocytosis, between the initial and second diagnoses. However, some typical features of the initial Ph^−^ MPN, for example, large and clustered megakaryocytes and grade 2 to 4 reticulin fibrosis, persisted after TKI treatment. Moreover, there was always a relatively long duration for Ph^−^ MPN transformation to CML with median duration of 4 years (range: 2–23 years). Only 4 patients initially presented with Ph^+^ CML and without *JAK2* V617F and *MPL* mutations. They were all given TKIs during the course of the diseases and responded well to the treatment with a constant decrease in *BCR-ABL1* fusion transcripts, which led to phenotypic changes. In turn, mutation screening showed positivity for *CALR* mutation and an association with ET or PMF phenotype. Indeed, the retrospective analysis indicated a high *CALR* mutation burden between initial CML diagnosis and secondary Ph^−^ MPN diagnosis, regardless of the *BCR-ABL1* transcript level. Control of the Ph^+^ clone took no more than 1 year of TKI treatment in these patients showing features of Ph^−^ MPN. In this cohort, only 1 patient (case 12 in Table 1) was diagnosed with CML with concomitantly detectable *CALR* mutation and *BCR-ABL1* fusion transcript.

### 3.2 Bone morrow features

The simultaneous presence of Ph^−^ MPN/Ph^+^ CML was evident in bone marrow morphology. For example, granulocytosis is the key CML feature, whereas unusually large and clustered megakaryocytes are characteristic of Ph^−^ MPN (but not CML). Indeed, assessment of megakaryocyte size can differentiate different MPN subtypes; for example, smaller megakaryocytes (micromegakaryocytes) are observed in CML, whereas large atypical forms of megakaryocytes are a typical feature of Ph^−^ MPN, according to a previous study.^[8]^ The presence of large atypical megakaryocyte forms with or without myelofibrosis in the CML bone marrow raises the possibility of simultaneous presence of Ph^−^ MPN and mutated *JAK2*/*CALR*/*MPL* should be searched for.^[5,9,10]^ In contrast, Ph^−^ MPN patients who are morphologically suspected to have CML but without cytogenetic confirmation should undergo further assessments such as fluorescent in situ hybridization and reverse-transcriptase polymerase chain reaction for gene mutation and fusion evolutions.

Moreover, myelofibrosis was a very common phenomenon in the bone marrow biopsy of this atypical MPN, that is, 11 of these 13 cases showing grade 2 to 3 reticulin fibrosis, which is consistent with another case series of concurrent *BCR-ABL1* fusion and *JAK2* V617F mutation.^[11]^

### 3.3 Clonal evolution

Occurrence of *CALR* driver mutation and *BCR-ABL1* fusion in the same patient could indicate the presence of different subclones and clonal evolution during disease progression and remission, mandating further investigation of *CALR* mutation and *BCR-ABL1* fusion in the same or different clones.

From his 11-year history of disease, early diagnostic specimens were no longer available for *CALR* mutation analysis and clonal evolution. However, based on his thrombocytosis and absence of the Ph chromosome at the first diagnosis, we speculate that our patient initially could have harbored *CALR* mutation, leading to the clinical and histologic manifestations of a Ph^−^ MPN, while the subsequent development of leukocytosis and acquisition of t(9;22)(q34;q11) suggested that CML evolved at a later point in time. However, we cannot rule out whether he initially harbored the *BCR-ABL1* clone with *CALR* mutation or only the negative *CALR* mutation.

To date, clonal analysis has been performed in only 1 case. Cabagnols et al^[12]^ assessed clonal hierarchy during evolution of the disease and showed that some of the original del52CALR clone was subjected to homozygosity transition, whereas the other clone led to *BCR-ABL1* fusion, suggesting that del52CALR and *BCR-ABL1* fusion were from the same clone and del52CALR was acquired before *BCR-ABL1* fusion. Although other cases did not have data from clonal analysis, they also discussed possible clonal heterogeneity and evolution in their patients.^[5,6,10–17]^ For instance, a case presented by Loghavi et al^[10]^ had fluorescence in situ hybridization data showing *BCR-ABL1* fusion signals in 78% of interphased cells in the initial sample, whereas the *CALR*-mutation allele occurred in 51% cells. Thus, they speculated that the *BCR-ABL1*-positive subclone could have arisen from a dominant clone with a heterozygous *CALR* mutation. Moreover, in case 5, the high *CALR* mutation level, concurrent with *BCR-ABL* fusion in almost 100% of the bone marrow cells, could be another strong argument in favor of the same clone that harbors both genetic alterations, which helped to shift the ET morphology to that of CML.^[13]^

However, several studies presented an opposing view, showing presence of 2 different clones or subclones, which can grow independently from each other.^[6,14–18]^ For example, a recent MPN study^[11]^ of concurrent *BCR-ABL1* fusion and *JAK2* mutation indicated that some JAK2^+^ MPN cells acquired *BCR-ABL1* fusions, whereas others had both genetic drivers at the initial diagnosis, suggesting that 2 independent clones arose from genetically unstable hematopoietic stem and progenitor cells and then competed with each other.

In summary, the majority of previously reported cases of Ph^+^ and *CALR*-mutated MPNs showed that *BCR-ABL1* fusion emerged from a previously *CALR*-mutated clone, and throughout the course of TKI treatment, the mutant *CALR* allele burden remained stable or progressively increased as the *BCR-ABL1* transcript declined, indicating that the Ph^+^/*CALR*-mutated clones were sensitive to TKIs, whereas Ph^−^/*CALR*-mutated clones were resistant to TKIs.

### 3.4 *CALR* subtype

Two main types of *CALR* mutations have been described in the literature to date: the 52-bp deletion (p.L367fs∗46, type 1 mutation) and the 5-bp insertion (p.K385fs∗47, type 2 mutation) in more than 80% of *CALR*-mutant MPNs.^[19]^ Since the discovery of these mutations, *CALR* mutations could be grouped into type 1 and type 2 *CALR* mutations in MPNs according to their predicted effects on the *C*-terminus of CALR.^[20]^ Pietra et al^[20]^ associated *CALR* type 1 mutation with a significant increase in the risk of myelofibrotic transformation among ET patients, whereas *CALR* type 2 mutations are preferentially associated with the ET phenotype, such as a low risk of thrombosis despite a very high platelet count and indolent disease progression, suggesting that these *CALR*-mutant subtypes correspond to different clinical phenotypes and outcomes. However, it will be interesting to know whether MPNs with concurrent *CALR* mutation and *BCR-ABL1* fusion show the same trend. Indeed, 10 of the 13 cases (76.9%) discussed here had type 1 or type 1-like *CALR* mutations and exhibited moderate myelofibrosis, whereas 2 of the other 3 cases harbored type 2 *CALR* mutation and showed an ET phenotype.^[6,13]^ Altogether, these 13 cases of MPNs more frequently had type 1 and type 1-like *CALR* mutations in PMF, which led to the evolution of a *CALR*-mutant MPN to Ph^+^ CML.

### 3.5 Treatment and response

The 13 patients were all treated with TKIs. After treatment, the *BCR-ABL1* transcript levels declined, although the mutant *CALR* allele burden remained stable or progressively increased. These data indicated that Ph^+^/*CALR*-mutated clones were sensitive to TKIs whereas the Ph^−^/*CALR*-mutated clones were not, suggesting that a combination of TKIs with cytoreductive medications, such as hydroxyurea and interferon, or with JAK inhibitor ruxolitinib, could effectively control disease progression. However, data concerning the timing, efficacy, and toxicity of TKI use in combination with other cytoreductive medications have not been presented previously, and more data are needed to confirm this notion. Moreover, case 9 received allogeneic hematopoietic stem cell transplantation and achieved complete remission with undetectable *CALR* mutation and *BCR-ABL1* fusion gene, suggesting that hematopoietic stem cell therapy is a useful option to cure this disease, especially in patients with treatment-related refractory disease.

However, given the paucity of data, it is still debatable whether the concurrent presence of *CALR* mutation indicates a better response to TKIs. Lewandowski et al^[21]^ speculated that simultaneous presence of different molecular alterations could complicate interpretation of the response of CML patients to TKIs. In their recent study, they demonstrated that TKI treatment was effective in CML patients with *JAK2* V617 and *CALR* mutations, and patients achieved major molecular response; however, 3 of 4 such patients still showed thrombocytosis and/or splenomegaly, indicating that TKIs could not control all clinical symptoms of this type of atypical MPN. In other studies, Fava and colleagues^[17]^ reported that 3 CML patients with simultaneous *JAK2* mutation and *BCR-ABL1* fusion only reached suboptimal responses to TKIs, whereas Boddu et al^[5]^ showed that 2 patients with JAK-V617-mutated CML rapidly progressed to a blast crisis after treatment with TKIs. In another study of 8 MPN patients with concurrent *BCR-ABL1* fusion and *JAK2* V617F/*CALR* mutations,^[6]^ only 4 patients achieved an optimal response after imatinib treatment, whereas 3 developed imatinib resistance, 2 of whom had *BCR-ABL1* kinase domain mutations. Taken together, these findings suggest that multiple gene alterations could complicate treatment selections and responses.

In the current case series, the response to TKIs among patients with a concurrent CALR mutation depended on the sequential appearance of CML and Ph^−^ MPN. For example, 4 patients with Ph^−^ MPN subsequent to CML all achieved major molecular response within 12 months of TKI treatment, whereas only 1 of the 5 patients with Ph^−^ MPN before CML achieved an optimal response within 12 months of TKI treatment. Ph^+^ clone could be the predominant clone and sensitive to TKI treatment. However, if *BCR-ABL1* fusion occurred after a long period of Ph^−^ MPN phase, such a clone might harbor other genetic aberrations, due to selective pressure from other drug treatments, for example, hydroxyurea, leading to an unfavorable treatment response. However, this interpretation needs to be confirmed by DNA sequencing of each clonal sample in the future.

## 4 Conclusion

In this case report and review of 12 additional cases with concurrent *CALR*-mutation and *BCR-ABL1* fusion, we highlighted the importance of integrated clinical, morphologic, and molecular genetic data for classifying atypical myeloid neoplasms for treatment and responses of patients. The coexistence of these gene alterations should be thoroughly investigated in cases of CML with persistent thrombocytosis, uncharacteristically advanced myelofibrosis, unusual megakaryocyte morphology, or failure to achieve an optimal response to primary therapy. So far, most studies have reported that *BCR-ABL1* fusion emerged from a previous *CALR*-mutated clone and that the Ph^+^/*CALR*-mutated clone, but not the Ph^−^/*CALR*-mutated clone, was sensitive to TKI treatment, suggesting that a combination of the TKIs with cytoreductive medications may be effective. Our current study lacks survival data; thus, there is a need for further investigation of the effects of these gene alterations and treatment options on the survival of MPN patients.

## Author contributions

CL designed the study and was involved in revising the manuscript. CW performed the data collection and was involved in writing the manuscript. RH performed all the molecular analysis for this patient. ZD performed the bone marrow aspiration and biopsy examination. MA provided more detailed information on case 4, respectively. All authors reviewed and approved the final version of the manuscript.

**Data curation:** Chunshui Liu, Manuel Abecasis, Cong Wang.

**Formal analysis:** Chunshui Liu, Cong Wang.

**Funding acquisition:** Cong Wang.

**Investigation:** Chunshui Liu, Ruiping Hu, Zhonghua Du, Cong Wang.

**Methodology:** Chunshui Liu, Ruiping Hu, Zhonghua Du, Cong Wang.

**Project administration:** Chunshui Liu, Cong Wang.

**Resources:** Cong Wang.

**Software:** Chunshui Liu, Ruiping Hu, Zhonghua Du, Manuel Abecasis, Cong Wang.

**Supervision:** Ruiping Hu, Cong Wang.

**Validation:** Cong Wang.

**Visualization:** Cong Wang.

**Writing – original draft:** Chunshui Liu.

**Writing – review & editing:** Cong Wang.
